# Negative Impact of Coronavirus Disease 2019 Pandemic on Gastric Cancer Care in Japan: A Tokushukai Real‐World Data Project 08 (TREAD 08)

**DOI:** 10.1002/jgh3.70285

**Published:** 2025-10-03

**Authors:** Rai Shimoyama, Yoshinori Imamura, Kiyoaki Uryu, Takahiro Mase, Masataka Taguri, Tadahisa Okuda, Megumi Shiragami, Yoshiaki Fujimura, Maki Hayashi, Hironobu Minami

**Affiliations:** ^1^ Department of General Surgery Shonan Kamakura General Hospital Kamakura‐shi Kanagawa Japan; ^2^ Cancer Care Promotion Center University of Fukui Hospital Fukui Japan; ^3^ Department of Hematology and Oncology University of Fukui Hospital Fukui Japan; ^4^ Department of Medical Oncology and Hematology Kobe University Graduate School of Medicine Kobe‐shi Hyogo Japan; ^5^ Department of Medicine and Oncology Yao Tokushukai General Hospital Osaka Japan; ^6^ Department of Breast Surgery Ogaki Tokushukai Hospital Gifu Japan; ^7^ Department of Health Data Science Tokyo Medical University Tokyo Japan; ^8^ Human Health Sciences Kyoto University Graduate School of Medicine Kyoto Japan; ^9^ Development Division Tokushukai Information System Inc. Osaka Japan; ^10^ Oncology Project Secretariat Mirai Iryo Research Center Inc. Tokyo Japan; ^11^ Cancer Center Kobe Kobe University Hospital Kobe‐shi Hyogo Japan

**Keywords:** cancer care, COVID‐19, gastric cancer, real‐world

## Abstract

**Aims:**

Concerns regarding the adverse impact of coronavirus disease 2019 (COVID‐19) on cancer care survival have been raised; however, clear evidence remains limited. Therefore, we aimed to investigate the influence of the COVID‐19 pandemic on gastric cancer management in Japan using real‐world data from the Tokushukai Real‐World Data project.

**Methods and Results:**

This retrospective cohort study was conducted across 46 Tokushukai Medical Group hospitals in Japan, identifying patients newly diagnosed with gastric cancer between January 2017 and December 2022. Patients with active double cancers or non‐epithelial tumors were excluded. We used data between January 2017 and March 2020 as the baseline (pre‐COVID‐19 period) to assess the changes in the number of diagnoses, screening detections, disease stage at diagnosis, and prognosis between April 2020 and December 2022 (mid‐COVID‐19 period). This study included 14 125 patients with 14 446 gastric cancer cases. Compared with the pre‐COVID‐19 period, the mid‐COVID‐19 period exhibited a 12% (95% confidence interval [CI]: 3%–20%) decrease in screening detections, a 9% (95% CI: 1%–18%) increase in metastatic stage detection, a 14% (95% CI: 7%–20%) decrease in curative surgery, and a 32% (95% CI: 19%–43%) decrease in radiation therapy. The analysis also revealed a 9.4% (95% CI: 2.0%–17.2%) increase in mortality in the mid‐COVID‐19 period compared with the pre‐COVID‐19 period.

**Conclusion:**

This nationwide, real‐world study provides robust evidence that COVID‐19 has reduced survival rates for Japanese patients with gastric cancer by disrupting diagnosis and treatment.

## 1 Introduction

In Japan, gastric cancer is the second most common cancer and the third leading cause of death [1], whereas globally, it ranks as the fifth most common cancer and the fourth leading cause of death [2]. Despite advancements in systemic treatments, the prognosis for advanced unresectable cases remains poor, with a 5‐year overall survival (OS) rate of 6.6%. However, early detection enables endoscopic treatment or radical surgery, with 5‐year OS rates in patients with localized and regional disease reaching 96.7% and 51.9%, respectively [3, 4]. Therefore, Japanese individuals over age 50 are recommended to undergo gastric fluoroscopy annually or gastroscopy every 2 years [5]. Additionally, *Helicobacter pylori* infection is a major risk factor for gastric cancer and is highly prevalent in Asia, Latin America, and Africa [6, 7, 8]. In Japan, serological screening that combines pepsinogen and *H. pylori* antibody is widely used to identify high‐risk individuals [9].

Since its emergence in Wuhan and subsequent global spread, the coronavirus disease 2019 (COVID‐19) has marked a pivotal shift towards a post‐pandemic era [10, 11]. In early 2020, healthcare systems worldwide were overwhelmed by COVID‐19 cases, necessitating rapid reallocation of human and financial resources to address the crisis. Consequently, several national cancer registries and multicenter retrospective studies reported decreased new cancer diagnoses, fewer early‐stage detections, and more advanced‐stage diagnoses in 2020 than in 2019 [12, 13, 14]. Notably, cancer diagnoses dropped sharply in April 2020, when COVID‐19 infection spread rapidly [13, 15]. In addition to the decreasing number of examinations, surgery, systemic therapy, and radiation therapy also decreased [16, 17].

Thus, cancer types that are highly dependent on routine screening for early detection, including gastric cancer, were most affected by the pandemic. In Japan, an analysis of cancer registry data reported a 12%–14% decrease in gastric cancer diagnoses and a 14% decrease in resections by 2020 [18, 19]. The number of individuals undergoing medical examinations, which markedly decreased following the outbreak of the pandemic, only slightly recovered in 2021 and has still not returned to pre‐pandemic levels [20]. Furthermore, a UK model study suggested an increase in mortality from breast, colon, esophageal, and lung cancers due to the delayed diagnosis caused by COVID‐19 [21]. These trends may be attributed to hindered access to timely diagnosis, reduced screening frequency, and interruptions in cancer treatment; however, direct evidence confirming this adverse effect on survival is lacking.

Therefore, in this study, we aimed to investigate the impact of the COVID‐19 pandemic on diagnosis, treatment outcomes, and prognosis of Japanese patients with gastric cancer using large‐scale real‐world data from the Tokushukai Medical Database.

## 2 Materials and Methods

### 2.1 Data Source

This nationwide Japanese retrospective cohort study was part of the Tokushukai Real‐World Data (TREAD) project [22], with minor modifications to the dataset (e.g., coverage period) made after the initial publication. As of December 2022, the Tokushukai Medical Group operated 71 hospitals nationwide (from Hokkaido to Okinawa) under a unified electronic medical record and coding system. Among these, 50 hospitals (70.0%) were eligible for the Diagnosis Procedure Combination system, and 51 (71.8%) had implemented a chemotherapy protocol system. A total of 46 hospitals had both systems and were thus included in this study. These comprised 3 nationally designated cancer hospitals, 3 prefectural designated cancer hospitals, and 30 general hospitals. This diversity in hospital functions and geographical distribution enhances the external validity of our results. During the COVID‐19 pandemic, no uniform, group‐wide restrictions were imposed; instead, each hospital proactively responded by admitting patients with COVID‐19 in accordance with local government policies.

Eligible patients included those with newly diagnosed gastric cancer at one of the 46 Tokushukai Medical Group hospitals (14 829 beds), identified using data integrated from the electronic medical record system (e‐Karte and Newtons2; Software Service Inc., Osaka, Japan), chemotherapy protocols (srvApmDrop; Software Service Inc., Osaka, Japan), national cancer registry data [23], and national health insurance claims data [24, 25] between 1 January 2017 and 31 December 2022. Patients with simultaneous and metachronous double cancers with a disease‐free interval of ≤ 5 years were excluded, except in cases of carcinoma in situ or intramucosal cancer that had been cured through local or endoscopic treatment. This operational definition was consistent with the multiple primary coding rules applied in the National Cancer Registry in Japan [23], which adhere to international cancer registry standards. Additionally, non‐epithelial tumors were excluded because their treatment approaches and prognoses differ substantially from epithelial gastric cancers.

Furthermore, the number of patients with COVID‐19 during the study period (collected via data tracked under the Infectious Diseases Control Law) was aggregated based on reports from the Ministry of Health, Labour, and Welfare to compare the infection status nationwide [26]. The classification of pandemic waves followed the timelines and data trends published by the National Institute of Infectious Diseases [27] and the Ministry of Health, Labour, and Welfare [26]. Since the first wave of COVID‐19 was marked as April–May 2020, the pre‐COVID‐19 period was defined as January 2017 to March 2020. While April 2020 and beyond was defined as mid‐COVID‐19, COVID‐19 waves 1–7 were defined as April–May 2020, July–September 2020, December 2020 to February 2021, April–May 2021, July–September 2021, January–March 2022, and July–September 2022. The mid‐COVID‐19 period comprised the on‐wave (waves 1–7) and off‐wave periods (between waves 1–7 and after wave 7).

### 2.2 Data Collection

Demographic data (age, sex, last confirmed survival date, and survival outcomes) were retrieved from medical records. Diagnostic information (diagnosis date, detection process, diagnostic facility, pathology, and stage) and treatment information (surgery, endoscopic procedures, radiotherapy, and systemic therapy) were sourced from hospital‐based cancer registry data. Cancer information is registered in the National Cancer Registry and simultaneously recorded in the Tokushukai Medical Database, which we utilized in this study. Cancer diagnosis dates were defined according to the National Cancer Registry standards: [23] pathological confirmation was prioritized; if unavailable, the earliest endoscopy or imaging date confirming malignancy was used. Survival status as of December 2023 was obtained from electronic medical records. Treatment information was further verified using the chemotherapy protocol system and health insurance claims data. Initial gastric cancer treatment was extracted from 30 days before to 90 days after diagnosis, based on prior studies showing that almost all patients initiate treatment within this timeframe [28]. Pathological staging was used when available; otherwise, clinical pathology was applied [29]. Tumor‐node‐metastasis components were identified, and all stages were aligned with the 8th edition of the Union for International Cancer Control [30].

Hospitals were classified as high‐volume if they enrolled at least 5% (674 cases) of the total cases and low‐volume if they enrolled fewer than 5% of the cases. Hospital types were categorized as government‐ and prefecture‐designated cancer treatment hospitals and general hospitals. Hospital urbanization was classified into megalopolis, urban, and rural areas based on the data collected by prefecture/secondary medical regions published by the Japan Medical Association Research Institute [31].

Curative treatment was defined as gastrectomy with lymph node dissection for stage 0–III patients, whereas endoscopic mucosal resection and endoscopic submucosal dissection were considered radical treatments for stage 0–I patients.

### 2.3 Statistical Analysis

Basic statistical analyses were conducted to summarize the distribution of patient background factors. Categorical variables are reported as calculated numbers and proportions, while continuous variables are reported as medians and ranges. Patients with non‐epithelial tumors were excluded from the diagnostic analysis, and those with active double cancers were excluded from the survival analysis.

The primary aim of our survival analysis was to describe OS across pandemic phases, rather than to identify independent prognostic factors. Therefore, univariable Cox regression was performed. Adjustment for covariates such as stage or hospital type, which are strongly associated with disease severity and healthcare access, could obscure the effect of the pandemic itself and could lead to overadjustment. For this reason, additional covariate adjustment was not undertaken.

The incidence ratio of patients during the pre‐COVID‐19 period (January 2017 to March 2020) and the mid‐COVID‐19 period (April 2020 to December 2022) was calculated for the incidence rate ratio (IRR) analysis. A Poisson regression model was used to analyze the differences between the two periods. Assuming a Poisson distribution for eligible patients, months within each COVID‐19 period (pre‐COVID‐19, mid‐COVID‐19, on‐wave, off‐wave) were treated as constants. The average number of patients per month, reflecting the number of incidences per unit time rather than strictly incidence rates based on person‐years, was assessed as an IRR. Confidence intervals (CIs) and *p* values for the IRR were estimated by converting to log‐rate ratios and using normal approximation. Dummy variables corresponding to months and COVID‐19 status were incorporated as adjustment factors in the Poisson regression model to account for seasonality in screening (e.g., fewer screenings during year‐end and New Year holidays, and at the end and beginning of fiscal years). The exponential transformation of the regression coefficients for COVID‐19 status was evaluated as the seasonally adjusted IRR. Monthly IRRs were derived from aggregated data; therefore, individual patient data‐based competing risk models are not applicable to these analyses.

A survival analysis was performed to compare mortality rates across different COVID‐19 periods. The study included individuals with recorded survival times who did not have concurrent active double cancer between 1 January 2017 and 31 December 2022. The endpoint was OS, defined as the time from a diagnosis to death from any cause, with survivors censored at the date of last contact. Notably, as OS captures all‐cause mortality, competing events do not arise. The results of the Kaplan–Meier (KM) curves and univariate Cox regression were considered the primary analyses. KM curves were used to compare the OS between the groups based on COVID‐19 status. Furthermore, COVID‐19 status was used as a prognostic factor in the Cox proportional hazards model. KM estimates were used to calculate mortality rates at 30 days, 90 days, 1 year, and 2 years for each COVID‐19 subgroup for secondary analyses. The CIs for these estimates were calculated using the variance in survival probabilities based on Greenwood's formula.

All analyses were conducted using the statistical software R (version 4.0.3, Foundation for Statistical Computing, Vienna, Austria). Two‐sided statistical tests were conducted, and *p* < 0.05 was considered statistically significant.

## 3 Ethics Approval

This study was performed in line with the principles of the Declaration of Helsinki. This study's approval was obtained from the Institutional Review Board of Tokushukai Medical Group (Approval Number: TGE01427‐024).

## 4 Results

**FIGURE 1:**
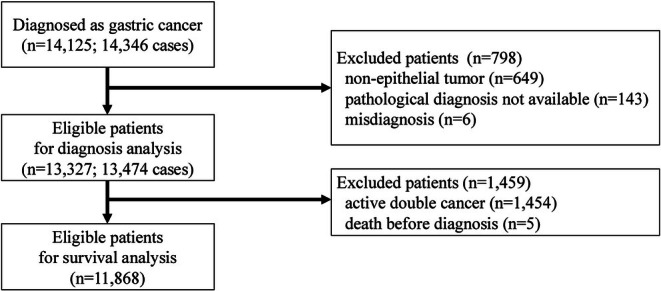
Patient flow diagram.

The study cohort included 14 125 patients with newly diagnosed gastric cancer between 2017 and 2022, resulting in 14 346 cases when accounting for multiple occurrences (Figure 1). Of these, 13 327 patients (13 474 cases) were included in the descriptive statistics, and 11 868 patients were included in the survival analysis. The median follow‐up period during the pre‐COVID‐19 period was 35.8 months, and during the mid‐COVID‐19 period was 12.6 months. Median follow‐up periods for patients during the on‐wave and off‐wave periods were 13.4 and 10.9 months, respectively.

### 4.1 COVID‐19 Pandemic in Japan

The status of the COVID‐19 pandemic in Japan is shown in Figure S1. A recurring trend of increasing COVID‐19 cases during waves and decreasing cases between waves was observed.

### 4.2 Patient Characteristics

In total, 13 327 patients (13 474 cases) were newly diagnosed with gastric adenocarcinoma. Detailed patient characteristics are provided in Table 1. Approximately 68% of the patients were men, and 55% of them were over 75 years old. Approximately half of the patients (48.4%) were diagnosed locally.

**TABLE 1:** Patient characteristics.

Characteristics	Context	Total cases (*N* = 13 474, %)	Pre‐COVID‐19 (*N* = 7248, %)	Mid‐COVID‐19 (*N* = 6226, %)	On the wave of COVID‐19 (*N* = 3383, %)	Off the wave of COVID‐19 (*N* = 2843, %)
Sex	Male	9222 (68.4)	5032 (69.4)	4190 (67.3)	2253 (66.6)	1937 (68.1)
	Female	4252 (31.6)	2216 (30.6)	2036 (32.7)	1130 (33.4)	906 (31.9)
Age (at diagnosis)	Median (range)	76 (17–103)	75 (17–102)	77 (24–103)	77 (25–103)	77 (24–100)
	≥ 75	7360 (54.6)	3794 (52.3)	3566 (57.3)	1946 (57.5)	1620 (57.0)
Background of diagnosis	Screening	1697 (12.6)	974 (13.4)	723 (11.6)	388 (11.5)	335 (11.8)
	Non‐screening	10 976 (81.5)	5805 (80.1)	5171 (83.1)	2815 (83.2)	2356 (82.9)
	Unknown	801 (5.9)	469 (6.5)	332 (5.3)	180 (5.3)	152 (5.3)
Histology	Intestinal	8116 (60.2)	4343 (59.9)	3774 (60.6)	2059 (60.9)	1715 (60.3)
	Diffuse	3530 (26.2)	1905 (26.3)	1625 (26.1)	892 (26.4)	733 (25.8)
	Mixed	117 (0.9)	53 (0.7)	64 (1.0)	34 (1.0)	30 (1.1)
	Indeterminate	1711 (12.7)	948 (13.1)	763 (12.3)	398 (11.8)	365 (12.8)
Primary site of disease	Gastric	12 350 (91.7)	6652 (91.8)	5698 (91.5)	3100 (91.6)	2598 (91.4)
	Esophagogastric junction	1124 (8.3)	596 (8.2)	528 (8.5)	283 (8.4)	245 (8.6)
Stage	Local	6524 (48.4)	3541 (48.9)	2983 (47.9)	1612 (47.7)	1371 (48.2)
	Regional	2186 (16.2)	1208 (16.7)	978 (15.7)	543 (16.1)	435 (15.3)
	Metastatic	2531 (18.8)	1315 (18.1)	1216 (19.5)	651 (19.2)	565 (19.9)
	Not available	2233 (16.6)	1184 (16.3)	1049 (16.8)	577 (17.1)	472 (16.6)
Hospital scale	High‐volume hospital (≥ 674 = 5%)^a^	4910 (36.4)	2723 (37.6)	2186 (35.1)	1176 (34.8)	1010 (35.5)
	Low‐volume hospital (< 674)	8564 (63.6)	4524 (62.4)	4040 (64.9)	2207 (65.2)	1833 (64.5)
Hospital type	Government‐designated cancer hospital	2312 (17.2)	1248 (17.2)	1064 (17.1)	586 (17.3)	478 (16.8)
	Prefecture‐designated cancer hospital	2381 (17.7)	1297 (17.9)	1084 (17.4)	588 (17.4)	496 (17.4)
	General hospital	8781 (65.2)	4703 (64.9)	4078 (65.5)	2209 (65.3)	1869 (65.7)
Hospital urbanization	Megalopolis	7569 (56.2)	4112 (56.8)	3457 (55.5)	1883 (55.7)	1574 (55.4)
	Urban	5403 (40.1)	2881 (39.7)	2522 (40.5)	1383 (40.9)	1139 (40.1)
	Rural	502 (3.7)	255 (3.5)	247 (4.0)	117 (3.4)	130 (4.5)

^a^ High‐volume hospitals were defined as those enrolling ≥ 674 cases (top 5% of hospitals).

### 4.3 Trends in Case Registrations

**FIGURE 2:**
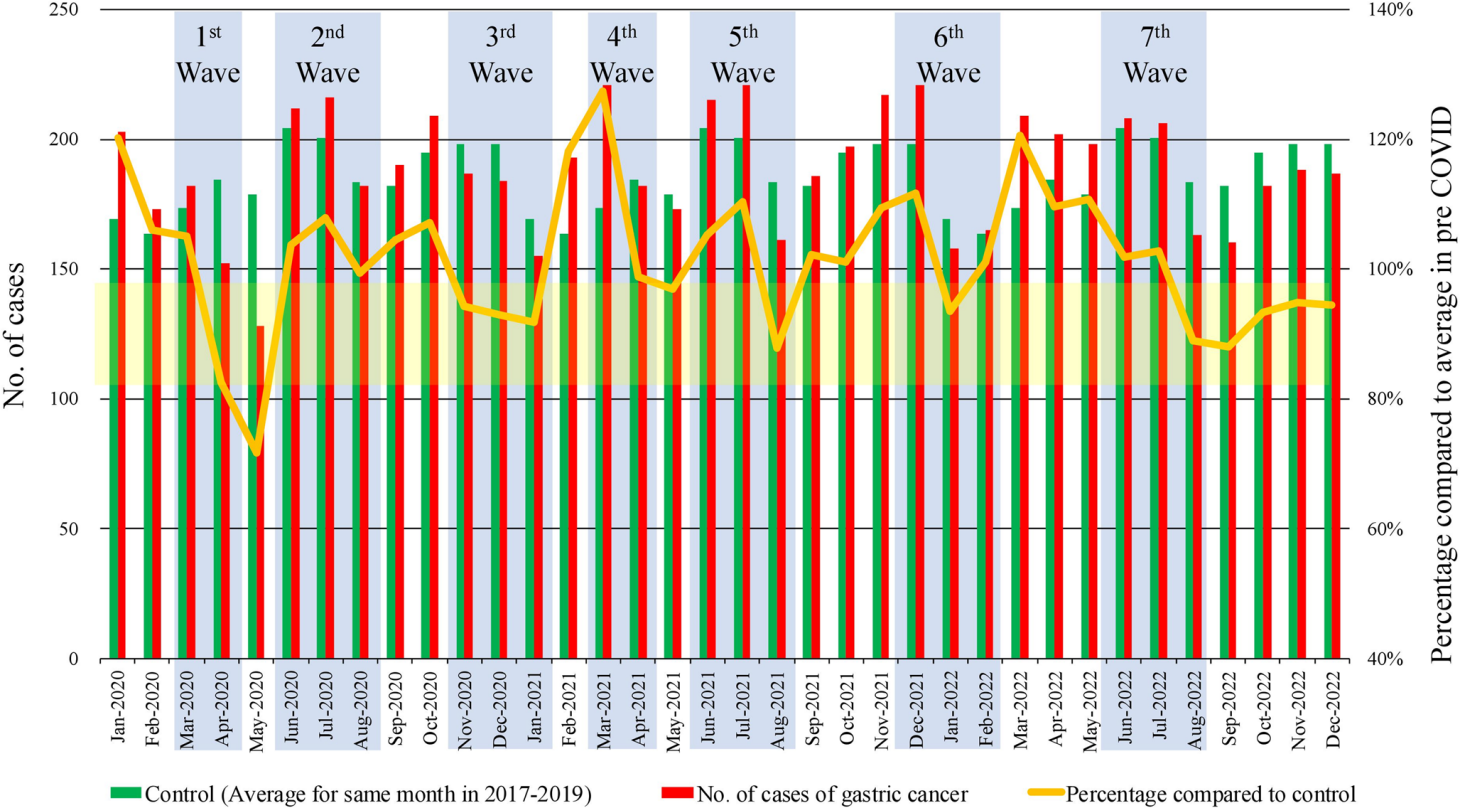
Trends in the number of gastric cancer cases in this cohort study. The ratio (yellow line) of the monthly average from 2017 to 2019 (green axis) to the actual number of diagnoses per month from January 2020 to December 2022 (red axis).

The monthly number of gastric cancer diagnoses between January 2020 and December 2022 is shown in Figure 2. Contrary to the trend observed in the number of patients with COVID‐19, gastric adenocarcinoma cases consistently decreased during pandemic waves, with a significant drop during the first wave and an increase between waves.

### 4.4 Trends in Initial Cancer Treatment in Gastric Cancer Cases

The initial treatments for gastric cancer are summarized in Table 2. Approximately half of the patients (47.5%) underwent surgery or endoscopic procedures with curative intent. No significant differences were observed between diagnosis and treatment across different periods.

**TABLE 2:** Details of initial cancer treatment.

Treatment		Total cases (*N* = 13 474^a^, %)	Pre‐COVID‐19 (*N* = 7248, %)	Mid‐COVID‐19 (*N* = 6226, %)	On the wave of COVID‐19 (*N* = 3383, %)	Off the wave of COVID‐19 (*N* = 2843, %)
Surgery		3714 (27.6)	2125 (29.3)	1589 (25.5)	865 (25.6)	724 (25.5)
Intent^b^	Curative	2961 (79.7)	1710 (80.5)	1251 (78.7)	675 (78.0)	576 (79.6)
	Palliative	597 (16.1)	320 (15.1)	277 (17.4)	151 (17.5)	126 (17.4)
	Unknown	156 (4.2)	95 (4.5)	78 (4.9)	39 (4.5)	39 (5.4)
Techniques^b^	Open	1892 (50.9)	1157 (54.4)	735 (46.3)	411 (47.5)	324 (44.8)
	Laparoscopic	1664 (44.8)	873 (41.1)	791 (49.8)	415 (48.0)	376 (59.1)
	Others	2 (0.1)	0 (0.0)	2 (0.1)	0 (0.0)	2 (0.3)
	Unknown	156 (4.2)	95 (4.5)	61 (3.8)	39 (4.5)	22 (3.0)
Procedure^b^	Total gastrectomy	915 (24.6)	549 (25.8)	366 (23.0)	201 (23.2)	165 (22.8)
	Distal gastrectomy	2188 (58.9)	1252 (58.9)	936 (58.9)	512 (59.2)	424 (58.6)
	Proximal gastrectomy	160 (4.3)	81 (3.8)	79 (5.0)	35 (4.0)	44 (6.1)
	Partial resection	15 (0.4)	10 (0.5)	5 (0.3)	4 (0.5)	1 (0.1)
	Palliative surgery	280 (7.5)	138 (6.5)	142 (8.9)	74 (8.6)	68 (9.4)
	Unknown	156 (4.2)	95 (4.5)	61 (3.8)	39 (4.5)	22 (3.0)
Endoscopic procedure		4273 (31.7)	2229 (30.8)	2044 (32.8)	1092 (32.3)	952 (33.5)
Intent^c^	Curative	3436 (80.4)	1816 (81.5)	1620 (79.3)	863 (79.0)	757 (79.5)
	Palliative	776 (18.2)	385 (17.3)	391 (19.1)	212 (19.4)	179 (18.8)
	Unknown	61 (1.4)	28 (1.3)	33 (1.6)	17 (1.6)	16 (1.7)
Procedure^c^	EMR/ESD	3528 (82.6)	1853 (83.1)	1675 (81.9)	893 (81.8)	782 (82.1)
	Stent	115 (2.7)	52 (2.3)	63 (3.1)	26 (2.4)	37 (3.9)
	Hemostasis	567 (13.3)	294 (13.2)	273 (13.4)	154 (14.1)	119 (12.5)
	Other	63 (1.5)	30 (1.3)	33 (1.6)	19 (1.7)	14 (1.5)
Chemotherapy		2049 (15.2)	1094 (15.1)	955 (15.3)	531 (15.7)	424 (14.9)
Radiotherapy		332 (2.5)	205 (2.8)	127 (2.0)	70 (2.1)	57 (2.0)
Time from diagnosis to treatment (days; median, range)		23.0 (−30–90)	22.0 (−30–90)	23.0 (−26–90)	23.0 (−23–90)	23.0 (−26–90)

Abbreviations: EMR, endoscopic mucosal resection; ESD, endoscopic submucosal dissection.

^a^ The total number of patients in each category does not equal the total number of patients because patients who received more than one treatment and patients who did not receive any of the treatments are included.

^b^ Percentage of cases that underwent surgery.

^c^ Percentages of cases that underwent endoscopic procedure.

### 4.5 Comparison of Gastric Cancer Diagnosis and Treatment During the Pre‐COVID‐19 and Mid‐COVID‐19 Periods

Table 3 presents a comparison between the pre‐COVID‐19 and mid‐COVID‐19 pandemic periods regarding the number of patients and their ratios for gastric cancer diagnosis, screening detection, stage, and cancer treatment. The mid‐COVID‐19 period revealed a 12% (95% CI: 3%–20%) decrease in screening detection and a 9% (95% CI: 1%–18%) increase in distant metastasis cases compared with pre‐COVID‐19. Moreover, a 14% (95% CI: 7%–20%) decrease in curative surgery and a 32% (95% CI: 19%–43%) decrease in radiotherapy were observed in the mid‐COVID‐19 period. After adjusting for seasonality, significant decreases were observed in screening, curative surgery, and radiotherapy.

**TABLE 3:** Comparison between the pre‐COVID‐19 and mid‐COVID‐19 periods.

Characteristics	Pre‐COVID‐19	Mid‐COVID‐19	Incident rate ratio (Mid/Pre)		Seasonally‐adjusted incident rate ratio (Mid/Pre)	
	/Month	/Month	Mean (95% CI)	*p*	Mean (95% CI)	*p*
No. of patients with gastric cancer	185.85	188.67	1.02 (0.98–1.05)	0.383	1.01 (0.97–1.04)	0.751
No. of patients with gastric cancer detected through screening	24.97	21.91	0.88 (0.80–0.97)	0.008	0.88 (0.80–0.97)	0.009
No. of patients with localized disease	90.79	90.39	1.00 (0.95–1.05)	0.859	0.99 (0.94–1.04)	0.598
No. of patients with metastatic disease	33.72	36.85	1.09 (1.01–1.18)	0.026	1.08 (1.00–1.17)	0.057
No. of patients who underwent curative surgery	43.85	37.91	0.86 (0.80–0.93)	0.000	0.85 (0.79–0.92)	0.000
No. of patients who underwent curative endoscopic procedure	46.56	49.09	1.05 (0.99–1.13)	0.122	1.04 (0.98–1.12)	0.205
No. of patients who underwent chemotherapy	37.79	35.33	0.93 (0.87–1.01)	0.086	0.93 (0.86–1.00)	0.056
No. of patients who underwent radiotherapy	8.21	5.58	0.68 (0.57–0.81)	0.000	0.68 (0.57–0.82)	0.000

Abbreviation: CI, confidence interval.

A similar comparison of the pre‐COVID‐19, on‐wave, and off‐wave periods is presented in Online Resources 1 and 2. During the on‐wave period, the number of gastric cancer diagnoses, localized cases, curative surgeries, systemic therapy sessions, and radiotherapy decreased significantly.

In contrast, during the off‐wave period, gastric cancer diagnoses and localized cases increased, along with the frequency of endoscopic procedures. However, the number of detection screenings, curative surgeries, systemic therapy, and radiotherapy sessions did not return to pre‐COVID‐19 levels. Notably, radiotherapy remained 33% (95% CI: 14%–48%) lower in the mid‐COVID‐19 period than that in the pre‐COVID‐19 period, while metastatic disease detection increased by 20% (95% CI: 8%–32%), even during the off‐wave period.

### 4.6 Overall Mortality in Gastric Cancer Cases

**FIGURE 3:**
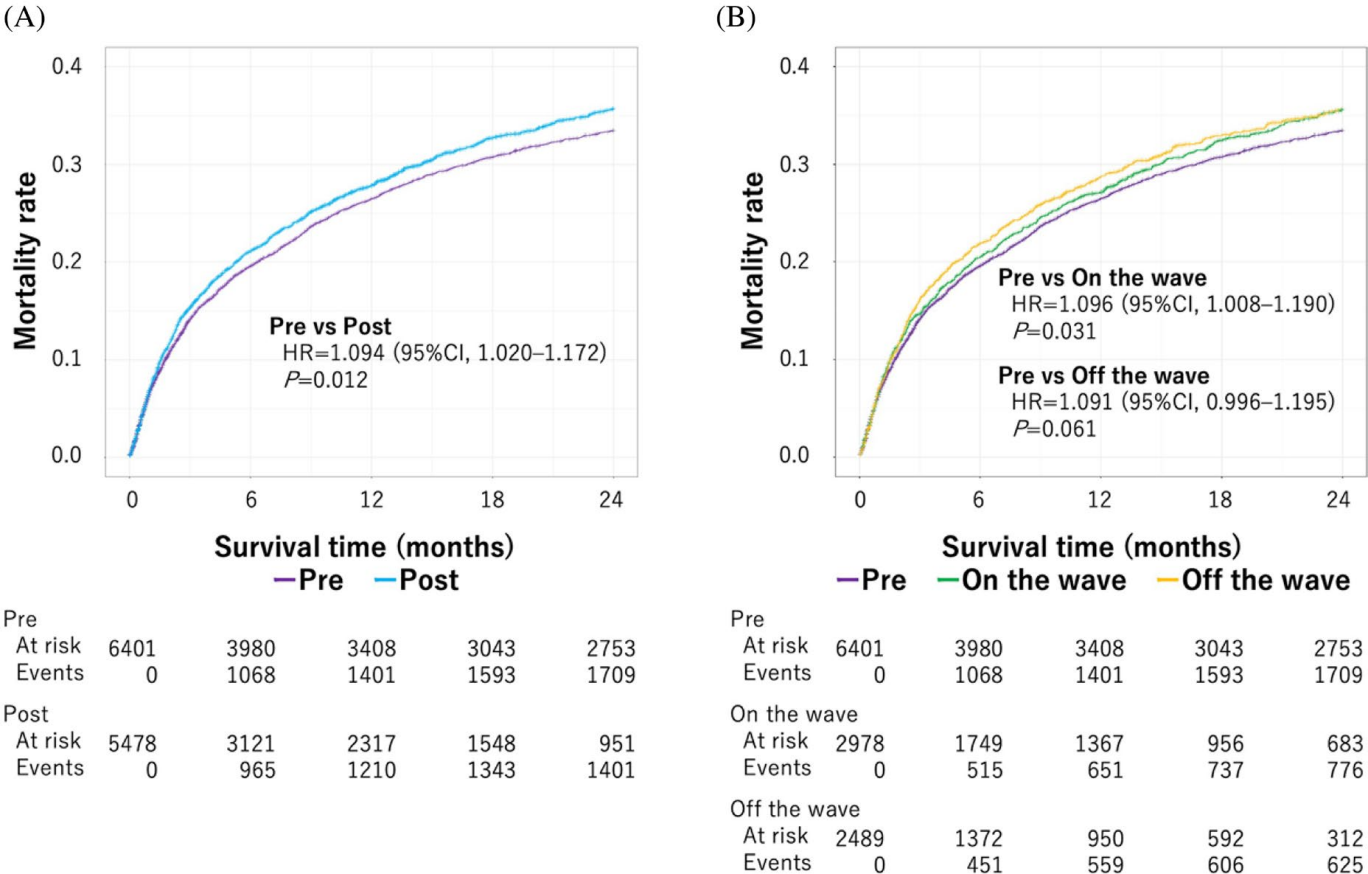
Kaplan–Meier curves of mortality in gastric adenocarcinoma cases. (A) Pre‐COVID‐19 period versus mid‐COVID‐19 period. (B) On‐wave period versus off‐wave period.

KM curves for mortality in gastric cancer cases (*n* = 11 868) are demonstrated in Figure 3. Patients diagnosed mid‐COVID‐19 had significantly worse mortality rates compared with those diagnosed during pre‐COVID‐19 (hazard ratio [HR] = 1.094; 95% CI: 1.020–1.172, *p* = 0.012). Similarly, the pre‐COVID‐19, on‐wave, and off‐wave periods were also compared, and a significant increase in on‐wave mortality was observed compared with that during pre‐COVID‐19 (HR = 1.096; 95% CI: 1.008–1.190, *p* = 0.031). Worsening mortality rates were also observed when comparing the pre‐COVID‐19 and off‐wave periods (HR = 1.091; 95% CI: 0.996–1.195, *p* = 0.061). The 30‐day, 90‐day, 1‐year, and 2‐year OS mortality rates are shown in Table 4. The OS rates during the mid‐COVID‐19 period were higher than those during the pre‐COVID‐19 period; however, these differences were not statistically significant.

**TABLE 4:** Mortality rate.

Mortality rate	Pre‐COVID‐19 (*N* = 6401)	Mid‐COVID‐19 (*N* = 5467)	On the wave of COVID‐19 (*N* = 2978)	Off the wave of COVID‐19 (*N* = 2489)
30‐day	6.6%	6.9%	6.7%	7.1%
(95% CI)	(6.0–7.2)	(6.2–7.6)	(5.7–7.6)	(6.0–8.1)
90‐day	14.1%	15.2%	14.6%	15.9%
(95% CI)	(13.1–15.0)	(14.2–16.2)	(13.3–16.0)	(14.3–17.5)
1‐year	26.5%	27.8%	27.1%	28.7%
(95% CI)	(25.3–27.7)	(26.5–29.2)	(25.3–28.9)	(26.6–30.8)
2‐year	33.5%	35.7%	35.6%	35.6%
(95% CI)	(32.1–34.8)	(34.1–37.3)	(33.5–37.8)	(33.1–38.1)

Abbreviation: CI, confidence interval.

## 5 Discussion

In Japan, gastric cancer cases have consistently decreased since the 2010s, with a year‐on‐year decline of 1.3%–3.8% reported between 2016 and 2019 [1]. However, in this study, the mid‐COVID‐19 period showed a 12% decrease in screening detection, a 9% increase in metastatic stage detection, a 14% decrease in curative surgery, and a 32% decrease in radiation therapy compared with the pre‐COVID‐19 period. Furthermore, these adverse effects were associated with a 9.4% increase in mortality. Thus, the change in the number of patients pre‐ and mid‐COVID‐19 likely exceeds the change estimated from the typical annual decline. In addition, an 11.8% decrease was observed in gastric cancer cases between 2019 and 2020 [1], likely due to reduced consultations during COVID‐19, although this is difficult to determine precisely without 2021 data.

All diagnoses and treatments decreased during the on‐wave period compared with the pre‐COVID‐19 period, except for the detection of metastatic diseases, which were presumed to be symptomatic. In the off‐wave period, a general increase was observed during the pre‐COVID‐19 period. However, the screening, curative surgery, or systemic therapy rates did not recover compared with those in the pre‐COVID‐19 period. Moreover, it may be difficult to make accurate comparisons between the on‐wave and off‐wave periods, as some patients are thought to have been affected by the waves in diagnosis but not in treatment, with other patients straddling both periods.

Several studies have investigated the negative impact of COVID‐19 on cancer diagnosis and treatment. An Italian study showed changes in the diagnoses of lung, colorectal, and breast cancers in 2019 before the onset of COVID‐19 compared to those in 2020, when COVID‐19 began. Particularly, the newly diagnosed lung cancer cases showed a decreasing trend [11], while the newly diagnosed colorectal and breast cancer cases decreased significantly [13, 14]. A Canadian study comparing the pre‐COVID (September 2016 to March 2020) and pandemic (March 2020 to October 2021) periods reported a 7.8% reduction in cancer diagnoses, with a 35% reduction in week 1 of the pandemic and subsequent recovery that did not reach pre‐COVID‐19 levels [17]. Another report from the same period showed a 12% decrease in gastric cancer, 8.3% in colon cancer, and 8.6% in rectal cancer diagnoses [21]. Similarly, the number of gastric cancers diagnosed in this study did not decrease; however, the number of metastatic lesions detected increased, which is consistent with the findings from a previous study [15]. Although our data showed no decrease in diagnoses, the reported number of cases in Japan was notably lower than the decrease expected from the pre‐COVID period, as mentioned above, suggesting a similar situation to that in other countries.

The decrease in screening examinations may have influenced the decrease in diagnoses. A French study examining the differences in screening, diagnosis, and treatment between 2019 and 2020 found an 86% decrease in fecal occult blood testing for colorectal cancer and a 100% decrease in mammography for breast cancer screening between April 2019 and April 2020 [16]. Meanwhile, a report from the United States comparing data between 2019 and 2021 predicted decreases in breast cancer screening (6%), cervical cancer screening (15%), and prostate screening (10%), while colorectal cancer screening remained unchanged [32]. Furthermore, another US study reported that while endoscopic procedures decreased by 12%, home‐based fecal occult blood testing increased by 44% [32]. Subsequently, a systematic review of 39 studies reported a 46.7% decrease in breast cancer screening, a 44.9% decrease in colorectal cancer screening, and a 51.8% decrease in cervical cancer screening during the pandemic after March 2020 [19]. Likewise, Japan also reported a decrease in screening diagnoses based on cancer registry data. Compared to the 2016–2019 average, screening detection in 2020 was expected to be 24.3% lower for gastric cancer and 13.4% lower for colorectal cancer [20]. Another Japanese study on gastric cancer using cancer registry data showed a 27.0% decrease in screening detection and a 23.9% decrease in early‐stage cancer [18]. Additionally, a study reported that by 2021, gastric cancer screening by fluoroscopy would decrease by 23.3% and colorectal cancer screening by 7.3% [20]. Accordingly, a 12% decrease in screening detection was observed in this study, which is consistent with the findings of previous studies, despite the screening detection rate being low in this cohort (15%) and the number of total gastric cancer cases remaining unchanged.

The reduction in screening examinations likely affected the initial staging of cancer diagnoses. An Italian study showed a significant increase in stage IV lung cancer cases [12] and a significant decrease in early‐stage colorectal and breast cancer cases (stages I, II, and III) [13, 14]. In Japan, according to cancer registry data [20], a decrease in gastric cancer cases was observed from stage I to stage IV, with a 15.3% decrease in stage I cases and a 13.9% decrease in stage II cases. The same report also documented a 5.9% decrease was observed in stage I and a 4.6% decrease in stage II colorectal cancer cases. However, the previous study reported no decrease in localized‐stage gastric cancer and a 9% increase in metastatic gastric cancer, likely due to the longer mid‐COVID‐19 period in this study than those previously reported (March 2020 to December 2022). In contrast, our data revealed an increase in metastatic cases during the mid‐COVID‐19 period, not only during the on‐wave period but also during the off‐wave period. This was presumably due to the aforementioned decrease in screening detection and increase in symptomatic cases detected.

Additionally, our cohort demonstrated significant reductions in curative surgery and radiotherapy and a non‐significant reduction in systemic therapy during the mid‐COVID‐19 period compared to the pre‐COVID‐19 period, which is consistent with previous studies. A study using the Japanese Cancer Registry reported a 14.1%, 9.2%, and 9.3% statistically significant decrease in resection cases from 2016 to 2019 to 2020 for gastric and colon cancers, respectively [19]. In addition, a Japanese study on gastric cancer reported that the number of endoscopic, laparoscopic, and open surgeries significantly decreased, whereas the use of systemic therapy and radiotherapy showed no significant change during the COVID‐19 pandemic [17]. Although studies investigating the relationship between pandemic waves are rare, we found that the radiotherapy implementation rate during the off‐wave period did not return to the pre‐COVID‐19 levels in our cohort. Thus, efforts to minimize the contraction of cancer treatments are crucial, even during pandemic waves.

To date, studies on the impact of COVID‐19 on cancer care survival are limited. A United Kingdom population‐based modeling study reported that the COVID‐19 pandemic was predicted to increase breast, colon, and esophageal cancer mortality by 7.9%–9.6%, 15.3%–16.6%, and 5.8%–6.0%, respectively [21]. Shigenobu et al. [33] reported on 4877 gastric cancer cases in a single prefecture between 2018 and 2021, identifying a HR for death of 1.17 during the pandemic, which supports an increased risk of mortality.

Consistent with these findings, our real‐world study demonstrated a 9.4% increase in mortality among Japanese patients with gastric cancer during the mid‐COVID‐19 period compared to the pre‐COVID‐19 period. This occurred despite the approval of first‐line combination chemotherapy with an immune checkpoint inhibitor in Japan in November 2021, which reportedly improves survival outcomes. Although the causes of death included both cancer and COVID‐19, our study demonstrated that the decrease in screening diagnoses, increase in distant metastasis cases, and reduction in curative surgeries may have ultimately worsened mortality.

Nonetheless, this study had some limitations, such as missing data owing to patient transfers from unregistered hospitals, resulting in incomplete comprehensive treatment history and prognosis information in certain cases. In addition, the short follow‐up period for mid‐COVID cases limits any reference to the impact on long‐term survival. Furthermore, information on COVID‐19 variants and vaccination status at the patient level was not available in our dataset. However, nationwide data exhibit sequential Alpha–Delta–Omicron waves and the progressive rollout of vaccination beginning in 2021 [34]. These contextual factors should be considered when interpreting the observed changes in gastric cancer care, as they may have contributed to variations in healthcare system capacity and patient behavior during the pandemic.

However, for the first time, we provide concrete evidence of reduced survival directly caused by COVID‐19, supported by comprehensive data analysis on cancer treatment and survival linked to individual patient records, using the most recent data available up to December 2022. Consequently, further studies extending beyond 2023 are warranted, along with similar analyses for the recommended cancer screenings in Japan, such as those for colorectal and breast cancers.

In conclusion, our nationwide real‐world retrospective study demonstrated the significant negative impacts of the COVID‐19 pandemic on the diagnosis, treatment, and prognosis of Japanese patients with gastric cancer. The data from this study provide crucial insights into cancer screening and treatment during future pandemics.

## Consent

The authors have nothing to report.

## Conflicts of Interest

R.S. received speaker bureau fees/honoraria from Daiichi‐Sankyo, Ono Pharm, Taiho Pharma, and Chugai outside the scope of the submitted work. Y.I. received speaker bureau fees/honoraria from Bayer, Bristol‐Myers Squibb, Daiichi‐Sankyo, Pfizer, and Ono Pharm outside of the submitted work. H.M. received speakers' bureau fees/honoraria from Chugai, Daiichi‐Sankyo and Ono Pharm; research funding from Astellas‐Amgen Biopharma, Bayer, Bristol Myers Squibb, Chugai, Daiichi‐Sankyo, Incite, Lilly, Novartis, and Ono Pharm; and scholarship donations from Astelas, Asahikasei Pharma, Bayer, Chugai, Daiichi‐Sankyo, Eisai, Kyowa Kirin, Lilly, Ono Pharmaceutical, Shionogi, Taiho Pharma, and Takeda, outside of the submitted work. It is important to note that these organizations played no role in the study's design, conduct, or reporting. All other authors declare no conflicts of interest.

## Supporting information


**Figure S1:** Trends in the number of COVID‐19 patients in Japan. The actual number of COVID‐19 diagnoses per month (red line) and pandemic waves (blue belt) from January 2020 to December 2022 were collected through comprehensive follow‐up surveys conducted under the Infectious Diseases Act [25].

**Table S1:** Pre‐COVID‐19 and on‐wave COVID‐19.

**Table S2:** Pre‐COVID‐19 and off‐wave COVID‐19.

## Data Availability

The data that support the findings of this study are available from the corresponding author upon reasonable request.
