# Supplementary figures and images for: Negative Impact of Coronavirus Disease 2019 Pandemic on Gastric Cancer Care in Japan: A Tokushukai Real‐World Data Project 08 (TREAD 08)
Source: JGH Open. 2025 Oct 3;9(10):e70285. doi: 10.1002/jgh3.70285 (PMC12491932; doi:10.1002/jgh3.70285)

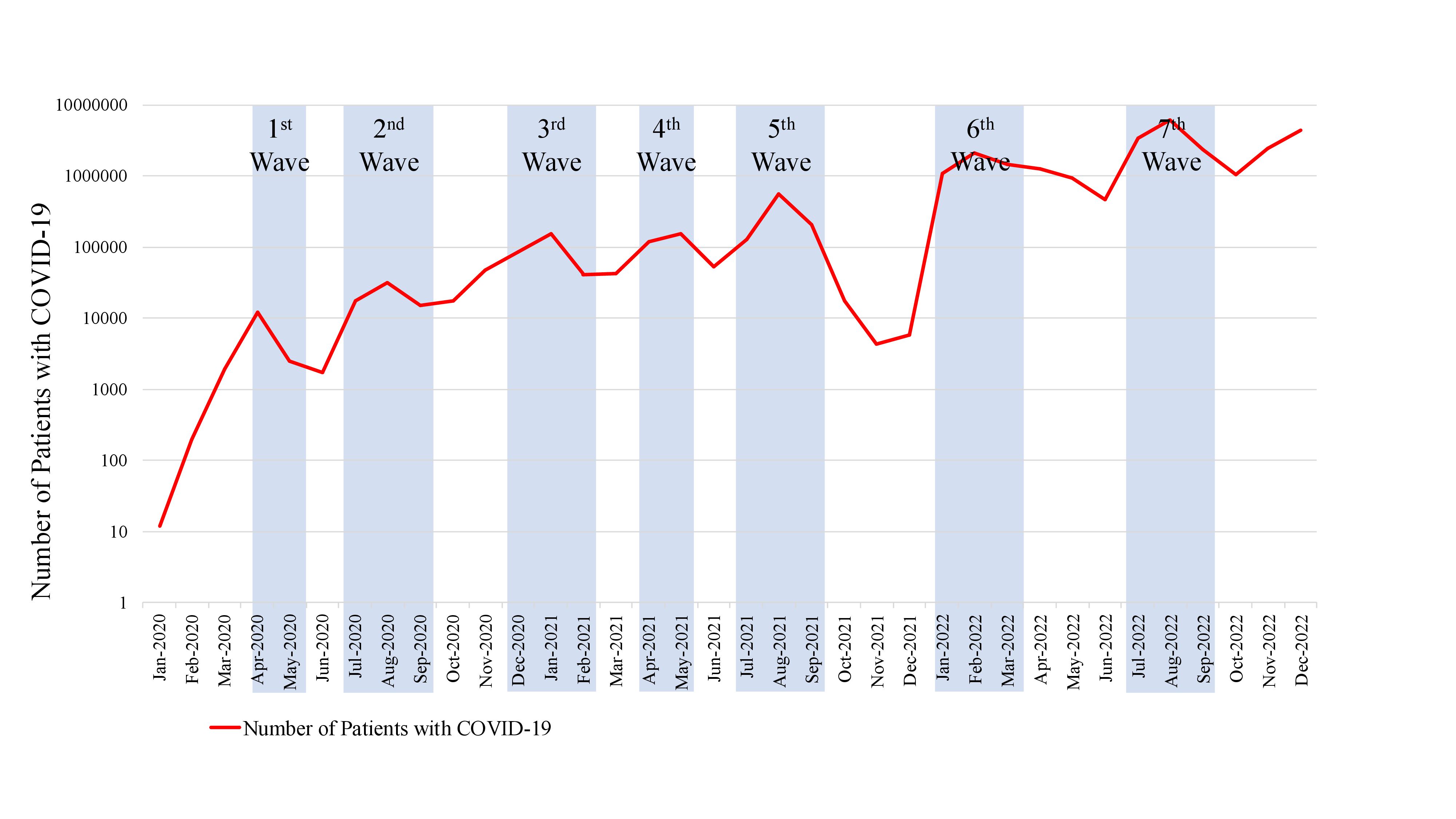

Supplement: Supplementary file 1 — Figure S1: Trends in the number of COVID‐19 patients in Japan. The actual number of COVID‐19 diagnoses per month (red line) and pandemic waves (blue belt) from January 2020 to December 2022 were collected through comprehensive follow‐up surveys conducted under the Infectious Diseases Act [25]. [file JGH3-9-e70285-s001.jpg]
