# Supplementary material for: Negative Impact of Coronavirus Disease 2019 Pandemic on Gastric Cancer Care in Japan: A Tokushukai Real‐World Data Project 08 (TREAD 08)
Source: JGH Open. 2025 Oct 3;9(10):e70285. doi: 10.1002/jgh3.70285 (PMC12491932; doi:10.1002/jgh3.70285)
Supplement: Supplementary file 2 — Table S1: Pre‐COVID‐19 and on‐wave COVID‐19. [file JGH3-9-e70285-s003.docx]

**Journal: Cancer Causes & Control**

Original article

**Negative impact of the coronavirus disease 2019 pandemic on gastric cancer care in Japan: A Tokushukai Real-world Data project (TREAD 08)**

Rai Shimoyama^1^, Yoshinori Imamura^2,3,4^, Kiyoaki Uryu^5^, Takahiro Mase^6^, Masataka Taguri^7^, Tadahisa Okuda^7,8^, Megumi Shiragami^9^, Yoshiaki Fujimura^9^, Maki Hayashi^10^, and Hironobu Minami^4,11^

^1^ Department of General Surgery, Shonan Kamakura General Hospital, Kamakura, Japan

^2^ Cancer Care Promotion Center, University of Fukui Hospital, Eiheiji, Japan

^3^ Department of Hematology and Oncology, University of Fukui Hospital, Eiheiji, Japan

^4^ Department of Medical Oncology and Hematology, Kobe University Graduate School of Medicine, Kobe, Japan

^5^ Department of Medicine and Oncology, Yao Tokushukai General Hospital, Yao, Japan

^6^ Department of Breast Surgery, Ogaki Tokushukai Hospital, Ogaki, Japan

^7^ Department of Health Data Science, Tokyo Medical University, Tokyo, Japan

^8^ Human Health Sciences, Kyoto University Graduate School of Medicine, Kyoto, Japan

^9^ Tokushukai Information System Inc., Osaka, Japan

^10^ Mirai Iryo Research Center Inc., Tokyo, Japan

^11^ Cancer Center Kobe, Kobe University Hospital, Kobe, Japan

**Corresponding author:** Yoshinori Imamura, M.D., PhD.

Cancer Care Promotion Center, University of Fukui Hospital

Address: 23-3 Matsuoka-Shimoaizuki, Eiheiji-cho, Yoshida-gun, Fukui, 910-1193, Japan

Phone: +81-776-61-3111

Fax: +81-776-61-8656

E-mail: [yimamura@u-fukui.ac.jp](mailto:yimamura@u-fukui.ac.jp)

Supplementary Table 1. Comparison between the pre-COVID-19 and on-wave COVID-19 periods

| Characteristics | Pre-COVID | On wave | Incident Rate Ratio  (On wave/Pre) | | Seasonally-adjusted  Incident Rate Ratio  (On wave/Pre) | |
| --- | --- | --- | --- | --- | --- | --- |
|  | /Month | /Month | Mean  (95% CI) | P-value | Mean  (95% CI) | P-value |
| No. of patients with gastric cancer | 185.85 | 178.05 | 0.96  (0.92–1.00) | 0.040 | 0.97  (0.93–1.01) | 0.125 |
| No. of patients with gastric cancer detected through screening | 24.97 | 20.42 | 0.82  (0.73–0.92) | 0.001 | 0.84  (0.74–0.95) | 0.007 |
| No. of patients with localized disease | 90.79 | 84.84 | 0.93  (0.88–0.99) | 0.024 | 0.94  (0.89–1.01) | 0.074 |
| No. of patients with metastatic disease | 33.72 | 34.26 | 1.02  (0.93–1.12) | 0.738 | 1.02  (0.92–1.12) | 0.747 |
| No. of patients who underwent curative surgery | 43.85 | 35.53 | 0.81  (0.74–0.89) | 0.000 | 0.81  (0.74–0.89) | 0.000 |
| No. of patients who underwent curative endoscopic procedure | 46.56 | 45.42 | 0.98  (0.90–1.06) | 0.548 | 1.00  (0.92–1.09) | 0.977 |
| No. of patients who underwent chemotherapy | 37.79 | 34.32 | 0.91  (0.83–1.00) | 0.040 | 0.92  (0.83–1.01) | 0.095 |
| No. of patients who underwent radiotherapy | 8.21 | 5.63 | 0.69  (0.55–0.85) | 0.001 | 0.71  (0.56–0.89) | 0.003 |

CI, confidence interval
