# Supplementary material for: Negative Impact of Coronavirus Disease 2019 Pandemic on Gastric Cancer Care in Japan: A Tokushukai Real‐World Data Project 08 (TREAD 08)
Source: JGH Open. 2025 Oct 3;9(10):e70285. doi: 10.1002/jgh3.70285 (PMC12491932; doi:10.1002/jgh3.70285)
Supplement: Supplementary file 3 — Table S2: Pre‐COVID‐19 and off‐wave COVID‐19. [file JGH3-9-e70285-s002.docx]

**Journal: Cancer Causes & Control**

Original article

**Negative impact of the coronavirus disease 2019 pandemic on gastric cancer care in Japan: A Tokushukai Real-world Data project (TREAD 08)**

Rai Shimoyama^1^, Yoshinori Imamura^2,3,4^, Kiyoaki Uryu^5^, Takahiro Mase^6^, Masataka Taguri^7^, Tadahisa Okuda^7,8^, Megumi Shiragami^9^, Yoshiaki Fujimura^9^, Maki Hayashi^10^, and Hironobu Minami^4,11^

^1^ Department of General Surgery, Shonan Kamakura General Hospital, Kamakura, Japan

^2^ Cancer Care Promotion Center, University of Fukui Hospital, Eiheiji, Japan

^3^ Department of Hematology and Oncology, University of Fukui Hospital, Eiheiji, Japan

^4^ Department of Medical Oncology and Hematology, Kobe University Graduate School of Medicine, Kobe, Japan

^5^ Department of Medicine and Oncology, Yao Tokushukai General Hospital, Yao, Japan

^6^ Department of Breast Surgery, Ogaki Tokushukai Hospital, Ogaki, Japan

^7^ Department of Health Data Science, Tokyo Medical University, Tokyo, Japan

^8^ Human Health Sciences, Kyoto University Graduate School of Medicine, Kyoto, Japan

^9^ Tokushukai Information System Inc., Osaka, Japan

^10^ Mirai Iryo Research Center Inc., Tokyo, Japan

^11^ Cancer Center Kobe, Kobe University Hospital, Kobe, Japan

**Corresponding author:** Yoshinori Imamura, M.D., PhD.

Cancer Care Promotion Center, University of Fukui Hospital

Address: 23-3 Matsuoka-Shimoaizuki, Eiheiji-cho, Yoshida-gun, Fukui, 910-1193, Japan

Phone: +81-776-61-3111

Fax: +81-776-61-8656

E-mail: yimamura@u-fukui.ac.jp

Supplementary Table 2. Comparison between the pre-COVID-19 and off-wave COVID-19 periods

| Characteristics | Pre-COVID | Off wave | Incident Rate Ratio  (Off wave/Pre) | | Seasonally-adjusted  Incident Rate Ratio  (Off wave/Pre) | |
| --- | --- | --- | --- | --- | --- | --- |
|  | /Month | /Month | Mean  (95% CI) | P-value | Mean  (95% CI) | P-value |
| No. of patients with gastric cancer | 185.85 | 203.7 | 1.09  (1.05–1.14) | 0.000 | 1.06  (1.01–1.11) | 0.020 |
| No. of patients with gastric cancer detected through screening | 24.97 | 23.93 | 0.96  (0.85–1.08) | 0.499 | 0.93  (0.81–1.06) | 0.285 |
| No. of patients with localized disease | 90.79 | 97.93 | 1.08  (1.01–1.15) | 0.017 | 1.05  (0.97–1.12) | 0.216 |
| No. of patients with metastatic disease | 33.72 | 40.36 | 1.20  (1.08–1.32) | 0.000 | 1.17  (1.04–1.30) | 0.006 |
| No. of patients who underwent curative surgery | 43.85 | 41.14 | 0.94  (0.85–1.03) | 0.187 | 0.91  (0.82–1.01) | 0.073 |
| No. of patients who underwent curative endoscopic procedure | 46.56 | 54.07 | 1.16  (1.07–1.26) | 0.001 | 1.11  (1.01–1.22) | 0.038 |
| No. of patients who underwent chemotherapy | 37.79 | 36.71 | 0.97  (0.88–1.07) | 0.571 | 0.94  (0.84–1.05) | 0.253 |
| No. of patients who underwent radiotherapy | 8.21 | 5.50 | 0.67  (0.52–0.86) | 0.002 | 0.65  (0.49–0.85) | 0.002 |

CI, confidence interval
